# P-954. Antibiotic Stewardship in Action: Reducing Vancomycin Use and VRE Incidence in a Community Hospital

**DOI:** 10.1093/ofid/ofaf695.1156

**Published:** 2026-01-11

**Authors:** Alex Ramos, Zachary I Pryor, James Newton

**Affiliations:** Washington Regional Medical Center/University of Arkansas for Medical Sciences, Fayetteville, AR; Washington Regional Medical Center/University of Arkansas for Medical Sciences, Fayetteville, AR; Washington Regional Medical Center, SPRINGDALE, Arkansas

## Abstract

**Background:**

Antibiotic Stewardship Programs (ASP) play a key role in curbing unnecessary antibiotic use and slowing the development of resistance. Vancomycin is a known contributor to the rise of vancomycin-resistant *Enterococcus* (VRE), a challenging healthcare-associated infection linked to longer hospital stays, higher costs, and fewer treatment options. Studies have demonstrated a direct correlation between vancomycin use and VRE emergence. ASPs have been shown to reduce VRE colonization by up to 70%. Rising VRE rates at Washington Regional Medical Center (WRMC) prompted a review of antibiotic use.

**Figure:**
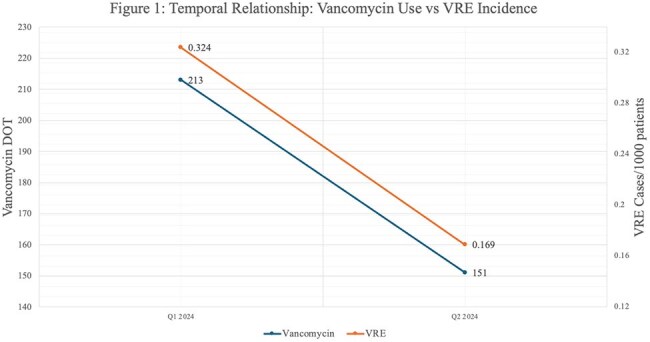

**Methods:**

From Q1 to Q4 of 2024, WRMC implemented a multipronged stewardship strategy to investigate and guide vancomycin use. Vancomycin utilization was tracked in Days of Therapy (DOT). The intervention included diagnostic stewardship with automatic PCR nasal screening when vancomycin was ordered for respiratory infections, rapid *S. aureus* PCR on gram-positive cocci blood cultures, and multiplex PCR assays to identify positive blood cultures. ASP engagement included daily ICU handshake stewardship rounds, daily virtual audits for medical-surgical patients, asynchronous staff education supported by evidence-based order sets, and direct pharmacist interventions featuring automatic pharmacokinetic dosing using AUC targets.

**Results:**

These combined stewardship efforts led to reduced vancomycin utilization, with vancomycin use declining from 18% to 14% of total antibiotic use, the lowest since ASP inception. Total use declined by 29% (213 DOT to 151 DOT). Over the same period, VRE rates decreased by 48% (0.324 to 0.169 cases per 1000 patient days). The trend is noted in Figure 1.

**Conclusion:**

Although direct causation cannot be established, the temporal correlation and magnitude of both changes are compelling and align with previous studies linking reduced vancomycin use to decreased VRE incidence. WRMC’s initiative highlights the value of multidisciplinary collaboration, rapid diagnostics, and pharmacist-led stewardship, offering a replicable model for stewardship programs aiming to improve patient safety and resistance trends.

**Disclosures:**

All Authors: No reported disclosures

